# A brief review of non-avian dinosaur biogeography: state-of-the-art and prospectus

**DOI:** 10.1098/rsbl.2024.0429

**Published:** 2024-10-30

**Authors:** Paul Upchurch, Alfio Alessandro Chiarenza

**Affiliations:** ^1^ Department of Earth Sciences, University College London, Gower Street, London WC1E 6BT, UK

**Keywords:** biogeography, dispersal, Dinosauria, Mesozoic, Pangaea, vicariance

## Abstract

Dinosaurs potentially originated in the mid-palaeolatitudes of Gondwana 245–235 million years ago (Ma) and may have been restricted to cooler, humid areas by low-latitude arid zones until climatic amelioration made northern dispersals feasible *ca* 215 Ma. However, this scenario is challenged by new Carnian Laurasian fossils and evidence that even the earliest dinosaurs had adaptations for arid conditions. After becoming globally distributed in the Early–Middle Jurassic (200–160 Ma), dinosaurs experienced vicariance driven by Pangaean fragmentation. Regional extinctions and trans-oceanic dispersals also played a role, and the formation of ephemeral land connections meant that older vicariance patterns were repeatedly overprinted by younger ones, creating a reticulate biogeographic history. Palaeoclimates shaped dispersal barriers and corridors, including filters that had differential effects on different types of dinosaurs. Dinosaurian biogeographic research faces many challenges, not the least of which is the patchiness of the fossil record. However, new fossils, extensive databasing and improved analytical methods help distinguish signal from noise and generate fresh perspectives. In the future, developing techniques for quantifying and ameliorating sampling biases and modelling the dispersal capacities of dinosaurs are likely to be two of the key components in our modern research programme.

## 1. Introduction

Historical biogeographic studies explore important questions regarding how abiotic factors (e.g. climate and sea level) have interacted with biotic factors (e.g. physiology, biomechanics and ecology) to shape organismal spatio-temporal distributions [1]. Thus, any holistic understanding of a group such as dinosaurs must include an appreciation of how taxa were distributed geographically, how and why distributions changed through time, and why some groups apparently departed from a more general pattern. Dinosaur evolution took place against the backdrop of the fragmentation of the supercontinent Pangaea and major fluctuations in sea level and climatic regimes (e.g. the ‘mid-Cretaceous’ thermal maximum) [2]. Changes in palaeogeography and palaeoclimates appear to have had a profound impact on dinosaurian evolution, yet not all taxa responded in the same way to these abiotic events: such discrepancies often hold the key to understanding ecological or physiological differences between particular groups. Aside from yielding insights into dinosaur evolution, the study of their palaeobiogeography has broader relevance. For example, many of the abiotic and biotic factors that affected this group are likely to have also impinged on other terrestrial Mesozoic tetrapods. Moreover, many extant organisms originated and radiated during the Mesozoic era, so an understanding of ancient biogeographic patterns and issues such as the impact of uneven fossil record sampling are relevant to the work of many neontologists.

Here, we outline the main ideas and debates in non-avian dinosaur biogeography, including their geographic origin, the tectonic and eustatic factors that have been implicated as controls on their distribution and recent developments relating to palaeoclimates and dinosaurian thermophysiology. We then examine some of the current challenges to understanding historical biogeographic patterns and processes, especially fossil record sampling, and highlight recently developed analytical approaches that open up new opportunities. We conclude by proposing some key questions and research topics that are likely to dominate this field in years to come.

## 2. Biogeographic origin

The earliest dinosaurs are known from Carnian age (early Late Triassic, *ca* 233−230 million years ago (Ma)) deposits in Argentina, Brazil, southern Africa and India [2–12]. This has given rise to the hypothesis that Dinosauria, and probably also its major subdivisions, originated and diversified in southern Gondwana during or before the Carnian and subsequently dispersed across the rest of Pangaea in the Norian (227 Ma onward) [3,6,7,11–24]. Although this southern Gondwana origin hypothesis (SGOH) remains the majority view among palaeobiogeographers, it is starting to be challenged by some recent studies that suggest that the prevalence of Carnian age southern Gondwanan dinosaurs represents uneven fossil record sampling and other ‘areas of origin’ (e.g. Laurasia) merit consideration [25–27]. For example, the carnivorous herrerasaurians have been variously placed as the sister taxon to theropods, sauropodomorphs, Saurischia or Dinosauria [27]. This group may have had a near-global distribution [10,11,28–30], opening up possible origins outside of southern Gondwana. Similarly, the enigmatic silesaurids might fill the stratigraphic and morphological ‘gap’ at the base of Ornithischia in the Late Triassic [18,27,31–37]: again, the fact that silesaurids are known from the Northern Hemisphere could potentially reshape our understanding of early dinosaur biogeography [27]. Finally, dinosaur tracks and theropod body fossils have recently been reported from the Carnian of Italy [38] and North America [39], respectively. These findings are contrary to the predictions of the SGOH, at least as initially formulated [13,21], though this hypothesis might be salvaged in a modified form if dinosaurs originated prior to the late Carnian. A somewhat earlier origin (*ca* 245−248 Ma) has been proposed based on Early–Middle Triassic footprints from Poland [17] and is consistent with late Carnian faunas comprising distinct lineages (e.g. Herrerasauria, theropods and sauropodomorphs), and the possibility that some dinosaurs had reached Laurasia by *ca* 230 Ma [38,39]. Thus, the modified SGOH envisages dinosaurs being restricted to southern Gondwana for the first 10−15 million years of their evolution, followed by diversification and dispersal in the late Carnian and early Norian [11].

## 3. Northward dispersals

**Figure 1.**
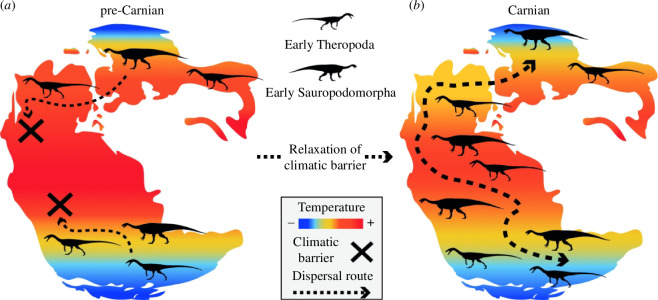
Impact of climate on early dinosaurian distributions. (*a*) High temperatures potentially generated an aridity belt at Pangaean low palaeolatitudes, acting as the primary barrier to northward dispersals, as envisaged by the southern Gondwanan origin hypothesis. (*b*) Under this scenario, the relaxing of these climatic barriers (black cross) during the Carnian Pluvial Event might have triggered an enhanced dispersal (dotted arrow) through Pangaea and consequent ecological release for early dinosaur lineages (see the main text for details and supporting references).

Whatever their starting point, dinosaurs were certainly widespread by the mid-Norian (*ca* 220−215 Ma), maintaining their presence in Gondwana and supposedly occurring for the first time in North America and Europe [1,3–5,7,15,40–42]. Closer inspection reveals a more nuanced picture, with herrerasaurians and theropods being the most widespread Norian dinosaurs and apparently reaching the northern hemisphere earlier, either in the mid-Norian (*ca* 219 Ma) [9] or in late Carnian [39]. In contrast, sauropodomorphs did not reach Europe and Greenland until *ca* 215 Ma and (along with ornithischians) were apparently absent from North America and Asia and low palaeolatitudes generally, until the very latest Triassic or Early Jurassic [2,4,9,18,43–45]. Several studies have, therefore, supported a ‘diachronous rise of dinosaurs’ hypothesis (DRDH), involving (i) an initial mid-palaeolatitude Gondwanan origin, (ii) restriction to these regions during the Carnian–early Norian, and (iii) mid-Norian northward ‘breakouts’ [9,15,45–47] (figure 1). This has been explained as the result of interactions between climatic zonation and early dinosaur palaeoecological or physiological requirements: in particular, the earliest dinosaurs may have been restricted to humid southern mid-palaeolatitudes during the Carnian because they could not tolerate the more arid and generally less stable conditions of the palaeotropics [45,47,48]. Such conditions suppressed plant productivity at lower palaeolatitudes, making them unsuitable for the large-bodied herbivorous sauropodomorphs [45]. Subsequently, *p*CO_2_ decreased through the Late Triassic–Early Jurassic (with notable dips occurring *ca* 215−212 Ma [9] and 206−202 Ma [44]), producing climatic ameliorations that made it more feasible for dinosaurs to cross lower palaeolatitudes, with the smaller bodied carnivores being the first to ‘breakout’ [9,45,47], though see [42].

The DRDH explains many aspects of early dinosaurian biogeography, but it can be challenged on a number of grounds and has undergone modification recently. For example, Griffin *et al*.’s [11] biogeographic models suggested that the wetter climates of the ‘Carnian Pluvial Event’ (234–232 Ma [38,48]) and decreases in *p*CO_2_ during the first 5−7 million years of the Norian allowed first theropods, and then sauropodomorphs, to disperse northward somewhat earlier than the original DRDH predicted. More serious challenges concern underlying assumptions about dinosaurian palaeoecology or physiology and plant productivity. After the Triassic, plant productivity remained relatively low in the palaeotropics, reflecting the persistence of harsher arid conditions (e.g. the 15–30° S Central Gondwanan Desert that stretched across West Gondwana during the Jurassic–Early Cretaceous) [49–60]. Dinosaurs occupied these lower palaeolatitudes later in their evolutionary history and must have crossed them repeatedly [2,61], raising the troublesome question, ‘Why were such conditions impassable to dinosaurs during the Carnian–early Norian but traversable during the rest of their evolutionary history?’ These issues can be clarified if we consider sauropodomorphs separately from other dinosaurs. Dunne *et al*. [62,63] demonstrated that early non-sauropodomorph dinosaurs (and their closest archosaurian relatives) occupied a wide array of climatic niches in terms of both mean annual temperatures and rainfall, perhaps reflecting ancestral mesothermic or endothermic metabolisms and adaptations useful for arid condition such as the production of uric acid rather than liquid urine [44]. Thus, it is difficult to argue that herrerasaurians and theropods were limited to cooler, humid conditions during their early evolution, and this could explain their earlier northward breakouts. In contrast, Late Triassic sauropodomorphs were restricted to cooler climates, potentially because of insufficient plant productivity, heat stress related to larger body size and/or competition from incumbent pseudosuchian herbivores in the palaeotropics [63]. In the Early Jurassic, sauropodomorphs (especially sauropods) expanded into warmer climatic zones [63], reflecting the disappearance of competitors (§4*a*) and/or adaptations that better equipped them for coping with heat stress (§5) and allowed a wider range of plant fodder [64–66].

## 4. Tectonics and sea level, vicariance and dispersal

### (a) Early Jurassic cosmopolitanism

Pangaea persisted as a largely coherent landmass during the Early and Middle Jurassic [67], leading many older studies to suggest that early dinosaurian faunas were cosmopolitan (i.e. similar in taxonomic composition) [68–75]. Recently, Button *et al*. [76] applied a novel biogeographic network approach (§6*b*) to quantify cosmopolitanism or endemism and found greater endemism in Laurasia during the Late Triassic (perhaps related to climatic barriers; §3), followed by increased cosmopolitanism in the Early Jurassic. This pattern, however, could not be observed for Gondwana, either because the shift from arid to more mesic conditions putatively responsible for increased cosmopolitanism at 30−60° N had not occurred in equivalent southern palaeolatitudes (e.g. the Central Gondwana Desert; §3) or because of fossil record sampling problems. Although climate may have facilitated increased Laurasian cosmopolitanism in the Early Jurassic, Button *et al*. [76] linked the latter to recovery from the end-Triassic mass extinction (ETE). Theropods and ornithischians may have diversified soon after the ETE, potentially taking advantage of niches left vacant by various non-dinosaurian groups such as phytosaurs and ornithischians [3–5,7,18,31,41,43,44,77–82], though see [45,63] for climate-based interpretations. Several important new lineages were present by the Early Jurassic, including tetanuran and ceratosaurian theropods [83] and the armoured thyreophoran ornithischians [84–86]. Sauropodomorphs had already become diverse in the late Norian–Rhaetian and appear to have been largely unaffected by the ETE, although the Early Jurassic witnessed the origin of eusauropods [3,4,66,87,88]. Indeed, the Early Jurassic was a time of major dinosaur diversification associated with elevated speciation rates [89,90], increases in body size [3,44,91], invasion of new ecological and climatic niches [63,92] and a much wider geographic distribution. The latter includes the first dinosaurs from Antarctica [93,94] and Asia [2,44,95] and the appearance of ornithischians and sauropodomorphs in North America [84,85,96]. At present, the direction of causality is not understood: did post-ETE vacant niches and/or climatic amelioration enable dinosaurs to disperse and experience conditions that prompted evolutionary innovations and diversification (adaptive radiation), or did dinosaurs acquire a suite of morphological, behavioural or physiological innovations for other reasons, enabling them to invade new niches and disperse (a ‘key innovation’)? These ideas are not mutually exclusive, and either way, they potentially explain an Early Jurassic expansion in dinosaur distributions and relative cosmopolitanism (at least in Laurasia).

### (b) Middle Jurassic–latest Cretaceous: reticulate biogeographic history

**Figure 2.**
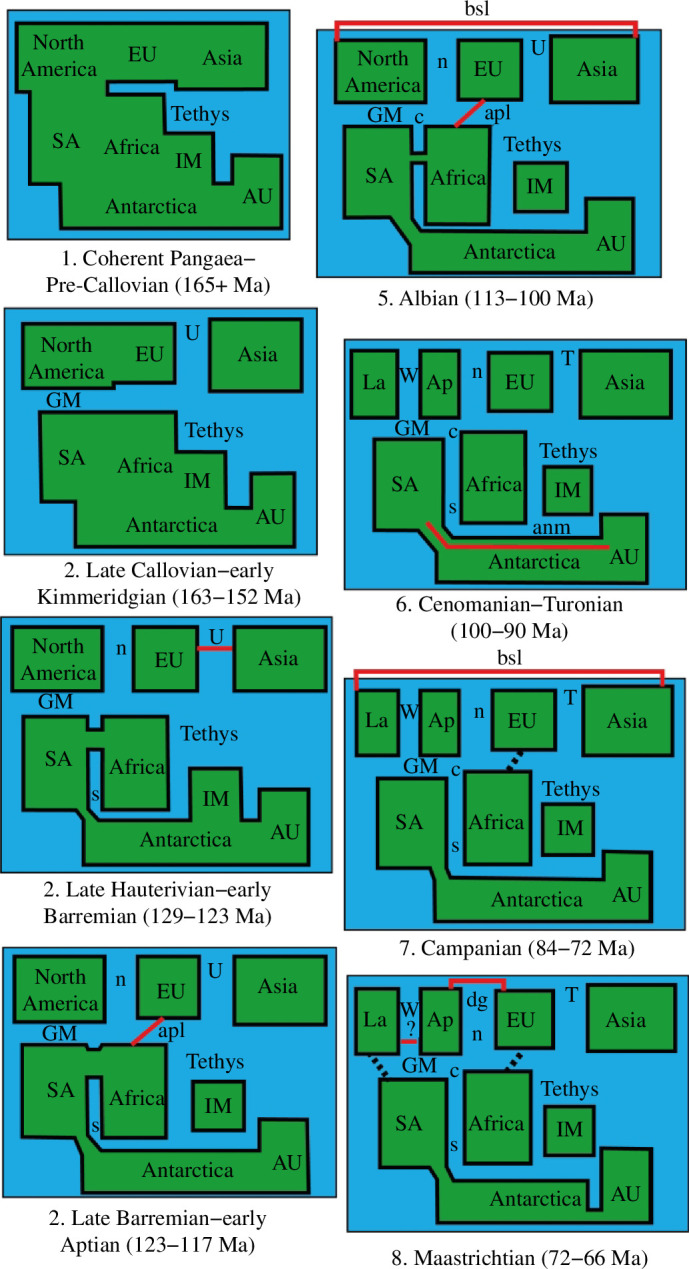
Schematic representation of changes in Pangaean palaeogeography during the late Mesozoic. Solid red lines denote ephemeral land connections; dashed black lines denote putative trans-oceanic/island-hopping dispersal routes. For more details and supporting references, see table 1 and [2]. Abbreviations: anm, Antarctic northern margin; Ap, Appalachia; apl, Apulian Landbridge; AU, Australasia; bsl, Bering Straits Landbridge; c, Central Atlantic; dg, De Geer Landbridge (links Greenland to Europe, so only applies if Appalachia was linked to Greenland at the same time); EU, Europe; GM, Gulf of Mexico; IM, Indo-Madagascar; La, Laramidia; n, North Atlantic; s, South Atlantic; SA, South America; T, Turgai Sea; U, Uralian Sea; W, Western Interior Seaway.

Dinosaurs continued to diversify during the Middle Jurassic, including the appearance of numerous groups that would later form major components of Late Jurassic and Cretaceous faunas, such as coelurosaurs (e.g. tyrannosauroids and Avialae), macronarian and diplodocoid neosauropods, and several ornithischian lineages (e.g. stegosaurs, ankylosaurs and ornithopods) [20,86,87,97–105]. Thus, many disparate lineages originated prior to Pangaean fragmentation (commencing *ca* 160 Ma [67]) and had an opportunity to achieve very widespread, or even global, distributions [2,58,83,87,106–110]. These widespread faunas were then divided by a series of tectonic and eustatic events during the Late Jurassic and Cretaceous, such as the separation of North and South America by the Gulf of Mexico, opening of the Atlantic, disintegration of East Gondwana and division of North America into Laramidia and Appalachia by the Western Interior Seaway (figure 2; table 1). Such a history predicts a classic vicariance scenario, whereby phylogenies should reflect the recency of connectedness of the geographic units inhabited [177]. There is evidence for vicariance, including endemism among Late Cretaceous faunas [178], statistically supported area relationships in phylogenies that conform to predictions derived from palaeogeography [106,132,161], maximum likelihood ancestral area estimations that support ‘best-fit’ models that incorporate vicariance [104,115,154,155] and phylogenetic network biogeographic analyses that demonstrate greater faunal similarities between geographic areas that were more recently in contact [133,179] (§6*b*). However, other studies have expressed scepticism about the importance of continent-scale vicariance, pointing to the supposedly cosmopolitan nature of Cretaceous dinosaurian faunas and anomalous phylogenetic relationships that are incongruent with palaeogeography [108–110,144,180–182] (§4*c*). To some extent, these debates reflect uncertainties created by ‘noise’ factors such as sampling failures (§6*a*). However, it is also probable that vicariance patterns have been partially obscured by other biogeographic phenomena such as dispersal and regional extinction, which in turn may set the scene for new vicariance patterns that overwrite older ones [106,132,161,183–185]. Substantial turnovers in dinosaurian communities occurred during the Jurassic or Cretaceous transition (*ca* 145−130 Ma) [86,161,186,187] and again in the early Late Cretaceous (*ca* 100−90 Ma) [100,138,139,150,161,163,188–191]. These events resulted in regional, or in some cases total, extinctions, which reshaped dinosaurian distributions and potentially increased regional endemism [22,83,107,109,111,180]. For example, rebbachisaurid sauropods died out completely, and all sauropod lineages apparently disappeared from North America, in the early Late Cretaceous [100,191–193].

**Table 1.** Summary of key landmass connection or disconnection events during the Middle Jurassic–latest Cretaceous that potentially impacted dinosaurian distributions. (N.B. These events, and their biogeographic consequences, are not always universally accepted—see [2,87,111] for further details and discussion.)

timing	event
late Callovian–Oxfordian (163−155 Ma)	separation of North and South America by the Gulf of Mexico, removing the last land connection between Laurasia and Gondwana [67,112,113]
late Callovian–early Kimmeridgian (163−152 Ma)	Uralian epicontinental sea separates Europe from Central and East Asia [67,87,114], associated with a distinct Chinese fauna (e.g. mamenchisaurid sauropods)
Kimmeridgian–?Early Cretaceous (157−130 Ma)	Gondwana may have been a coherent landmass, isolated from Laurasia, potentially resulting in vicariant origins of major clades such as Coelurosauria and Marginocephalia in Laurasia and Carcharodontosauria and Titanosauria in Gondwana [100,111,115–117]
late Kimmeridgian–early Tithonian (152−147 Ma)	land connections between North America and parts of western Europe [118] create a Euramerican dinosaurian fauna, including several shared genera or species (e.g. *Allosaurus* and *Stegosaurus*) [119–121]
Jurassic or Cretaceous transition (*ca* 145−130 Ma)	?separation of Gondwana into Samafrica (South America + Africa) and eastern Gondwana (Antarctica + Indo-Madagascar + Australasia). South America probably maintained a connection with eastern Gondwana, throughout much of the Cretaceous, via Patagonia-western Antarctic Peninsula [67,116,122,123]
early Berriasian (145−142 Ma)	marine regression removes Uralian Sea barrier between Europe and Central or East Asia [118]
Valanginian (139−133 Ma) and/or late Hauterivian–early Barremian (129−123 Ma)	land connections bridge proto-North Atlantic, allowing geodispersal of turiasaur sauropods and some ornithopods between Europe and North America [67,118,124,125], though see [2,126]
late Hauterivian–early Barremian (129−123 Ma)	marine regression removes Uralian Sea barrier between Europe and Central or East Asia [118], potentially enabling geodispersal of spinosaurid theropods, somphospondylan sauropods and ankylopollexian ornithopods from Europe to Asia [101,125,127–129] and oviraptorosaur theropods and ceratopsians from Asia to Europe [130,131]
late Barremian–Albian (123−100 Ma)	Apulian landbridge forms between Europe and North Africa, allowing geodispersal: for example, abelisauroid, compsognathid, carcharodontosaurid and spinosaurid theropods; rebbachisaurid and titanosaurian sauropods. Results in the formation of the ‘Euro-Gondwana’ biogeographic pattern in the Early Cretaceous [103,104,132–137]
Aptian–Albian (121−100 Ma)	sea level decreases remove Uralian Sea marine barriers between Europe and Asia, and the Bering Straits landbridge forms between Asia and North America. Potentially results in a coherent Laurasia and geodispersal [106,132] (see also [138–142])
early Aptian (119 Ma)	separation of Indo-Madagascar from northern margin of Antarctica isolates former region during the rest of the Cretaceous [2,116,143]
Albian or Cenomanian (*ca* 100 Ma)	final separation of Africa from South America via the opening of the central Atlantic [116,144–147]. Potentially results in vicariance (e.g. among titanosaurs [115])
late Albian–Turonian (105−90 Ma)	southward shift of more temperate climatic zone facilitated dispersal of titanosaurs from South America to Australasia via Antarctica [111,148,149]
late Albian–Campanian (105−72 Ma)	Western Interior Seaway separates North America into Laramidia and Appalachia (with a short-lived reconnection during the early Cenomanian) [150–152], potentially producing an endemic Appalachian fauna in the Coniacian–Campanian [153]
late Turonian–early Santonian (94−85 Ma)	sea level decrease forms a landbridge across the Turgai Sea, linking Europe and Asia. Potentially facilitates geodispersal of alvarezsauroids, therizinosauroids, oviraptorosaurs, dromaeosaurids, titanosaurs and hadrosauroids—mainly from Asia to Europe [115,154–158]
late Santonian–early Campanian (85−78 Ma)	sea level increase re-establishes the Turgai Sea as a barrier between Europe and Asia [111,159]
Campanian–Maastrichtian (84−66 Ma)	dispersal (via landbridge or island hopping) between North and South America, with titanosaurs moving north and hadrosaurs and ankylosaurs moving south [113,115,142,154,155,160]. N.B. Doubts have been expressed about this scenario [132,161,162]
Campanian (84−72 Ma)	Bering Strait landbridge re-forms, facilitating geodispersal between Laramidia and East Asia of multiple theropod lineages (e.g. alvarezsaurids, ornithomimids and tyrannosaurids), ceratopsids, ankylosaurids and some later-branching hadrosaurids, ‘refreshing’ the distinctive Asiamerican fauna [104,141,163–166]
mid-Campanian (78−76 Ma) and Campanian/Maastrichtian boundary (72 Ma)	sea level low-stands create land connections (or more probably facilitate island-hopping or trans-oceanic dispersal) between Europe and North Africa, resulting in hadrosaur and titanosaur dispersals [154,155,157,167–169]
mid-Campanian–early Maastrichtian (78−69 Ma)	Lambeosaurine hadrosaur lineages arrive in Europe, though it is not clear whether this involved dispersal from Asia, North America or both, with one or more dispersal events in the Campanian and Maastrichtian [155,170–172] (see ‘De Geer landbridge’ below)
Maastrichtian (72−66 Ma)	De Geer landbridge connects Greenland to northwestern Europe [164], potentially facilitating the dispersal of leptoceratopsids and lambeosaurines from North America into Europe [157,173], though see [2]
?late Maastrichtian (69−66 Ma)	a landbridge reconnects Laramidia–Appalachia, across the Western Interior Seaway, potentially facilitating geodispersal of ceratopsids into Appalachia [174,175], though see [176]

Although controversial, several studies have suggested that dinosaurs were capable of dispersing across marine ‘barriers’ via rafting or swimming, potentially modifying faunal compositions and partially overwriting vicariance patterns [155,194,195]. For example, Longrich *et al*. [155] reported the presence of the hadrosaur *Ajnabia* from the late Maastrichtian (69−66 Ma) of North Africa: the close phylogenetic relationships of *Ajnabia* with Laurasian taxa suggested that hadrosaurs reached Africa from Europe by crossing 500 km of the Tethys Ocean. Similar trans-Tethyan dispersals from Europe may also explain the presence of the titanosaurian sauropods *Mansourasaurus* and *Igai* in the latest Cretaceous of Egypt [154,167].

Finally, and most importantly, landmasses are as likely to become connected as they are to become disconnected, so the history of geographic units and the biotas they support are more accurately regarded as having network-like (‘reticulate’) histories rather than purely branching ones [183–185,196] (figure 2; table 1). This frequent overprinting potentially explains why putative vicariance patterns are manifested at low taxonomic levels (genera and species) [106,108,148], and so are difficult to identify without large datasets and powerful analytical methods. Essentially, the connection of two previously separate geographic areas allows biotic exchanges via ‘geodispersal’, and this creates new distributional patterns that cut across older ones [106,132,161,183–185]. Thus, we need to think of dinosaurian biogeographic history as a palimpsest of multiple (and often conflicting) signals laid down during different phases of Earth’s history [2,106,111].

### (c) Australia: a Cretaceous case study

Several studies have argued that mid-Cretaceous Australian dinosaurian faunas do not display the strong similarities with those in other parts of Gondwana, predicted by vicariance and palaeogeography [108,109,182]. Indeed, it has even been claimed that these Australian dinosaurs are more similar to those from Laurasia than Gondwana, leading to explanations based on long-distance trans-oceanic dispersal [197–199] or climatic zonation [108,109]. For example, Benson *et al*. [109] proposed that tyrannosaurs were present in Laurasia and Australia during the Late Cretaceous because they were more suited to the cooler and more humid conditions in these regions, whereas abelisaur theropods occupied the large predator niches in other parts of Gondwana because they preferred arid climates. However, new fossil discoveries and recent taxonomic and phylogenetic work have strengthened the evidence that mid-Cretaceous Australian dinosaurs are part of a wider Gondwanan vicariance pattern. In particular, there are close phylogenetic relationships between South American (and when available, Antarctic) and Australian dinosaurs, such as titanosaurian sauropods [111,148], noasaurine and megaraptorid theropods [161,200–202] and parankylosaurs [203,204]. This is reinforced by the phylogenetic network biogeographic analyses of Kubo [179], which demonstrated that Australian dinosaurs had their strongest links with faunas in South America and that Gondwanan areas generally had faunas that were more similar to each other than to those of Laurasia (especially Asiamerica) during the Late Cretaceous (table 1; see also [161]).

## 5. Impact of climate: latitudinal distributions and dispersal

**Figure 3.**
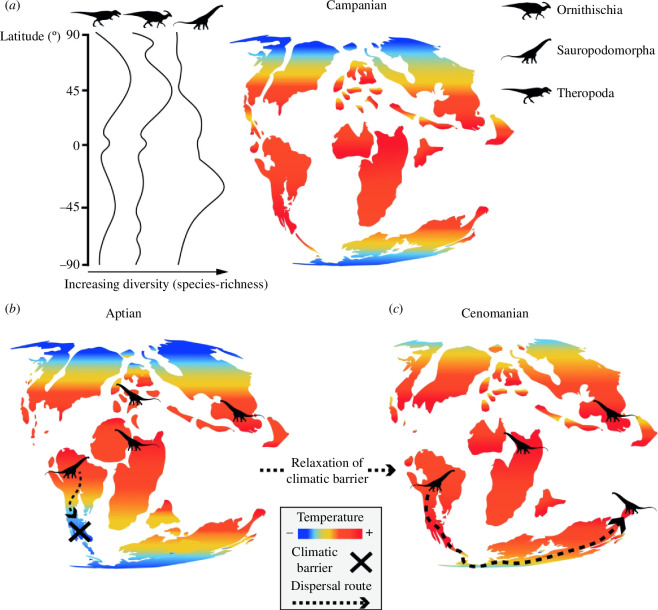
Dinosaurian palaeolatitudinal distribution and dispersal. (*a*) The latitudinal diversity pattern of non-avian dinosaurs during the better-sampled Late Cretaceous interval does not conform to a modern-style latitudinal diversity gradient, with only sauropod diversity potentially peaking at low latitudes, and mostly in the Southern Hemisphere. (*b*) The putative preference of sauropods for hotter, semi-arid zones may have meant that cooler climates formed barriers to their dispersal in some intervals such as the early Late Cretaceous. (*c*) Sauropod dispersal might have occurred from South America to Australia via Antarctica [111] during the Cenomanian hyperthermal (see main text for details and supporting references).

Today, the diversity of extant tetrapods peaks in the tropics and decreases toward the poles, a pattern known as the modern latitudinal biodiversity gradient [205,206]. However, Mesozoic dinosaurs often exhibited peak diversity at temperate palaeolatitudes [61,62]. Moreover, as noted in §3, there were differences in the climatic niches occupied by different dinosaurian groups, and this is manifested in their latitudinal distributions [44,61,62,207]. For example, Chiarenza *et al*. [207] analysed the relationships between palaeoclimatic parameters and dinosaurian distributions using habitat suitability modelling (HSM) and corrections for sampling biases. This study found that poor sampling was a problem for the Early–Middle Jurassic palaeotropics, particularly between 0 and 30° S (areas corresponding to the Sahara and Amazon today) but was minimal in the Cretaceous. Broadly speaking, Late Jurassic–Early Cretaceous peak diversity typically occurred around 20−40° N (Ornithischia, Theropoda) and 30° N (Late Jurassic sauropods) or at the equator (Early Cretaceous sauropods), with some variation between the Northern and Southern Hemispheres. By contrast, Late Cretaceous peak diversity occurred at 40−45° N (Ornithischia), 45° N and 40° S (theropods) and 30° S (sauropods; figure 3). Thus, the latitudinal distributions of sauropods seem to have differed from those of theropods and ornithischians, and peak diversities shifted further toward the temperate palaeolatitudes in the Late Cretaceous. An apparent difference between ornithischians + theropods and sauropods was reinforced by the HSM results, which suggested that the latter group was less tolerant of colder conditions (see also [63]). These differences probably reflect the complex interplay between palaeoecology, biomechanics, physiology, climate, palaeogeography and evolutionary contingency. Sauropods may have been able to tolerate high environmental temperatures via traits such as a bird-like respiratory system, long necks and tails for increased surface area and unique vascular systems, which facilitated shedding excess body heat [64,66]. Chiarenza *et al*. [92] suggested that sauropods had a unique physiology, with temperature regulation more like modern reptiles, thus restricting them to warmer climates. Interestingly, the Cenomanian–Turonian Thermal Maximum (94−91 Ma) supported diverse flora and savanna-like environments, favouring large primary consumers [208,209], and it is during this interval that sauropods achieved their greatest body masses [210,211]. On the other hand, lower plant productivity at polar latitudes, even under warmer global climates than today [52,53], may have made it impossible for sauropods to acquire enough fodder to support their gigantic bodies [92,111]. By contrast, ornithischians and theropods exhibited high diversity at approximately 40°−50° palaeolatitudes, particularly in the Northern Hemisphere, with some taxa apparently living at 70° or higher [44,149,207,212–214] (N.B. no sauropod has been found at palaeolatitudes higher than 66°, and high latitude instances are very rare [111,215,216].) Theropods and ornithischians probably possessed meso- or endothermic metabolisms and feather-like insulation, which enabled them to cope with colder conditions [44,63,207,217,218]. Biomechanical, isotopic and osteohistological evidence, combined with findings of nesting and perinate material, suggests that northern high-latitude theropods and ornithischians were adapted to Arctic winters and were resident there year-round (i.e. they did not undertake seasonal southward migrations) [213,214,219].

As noted above, sauropods and ornithischians were most diverse and abundant in Gondwana and Laurasia, respectively [2,61,207]. Such hemispherical asymmetries potentially reflect climatically driven habitat distributions, evolutionary contingency and biogeographic factors. For example, HSM suggests that the hotter and semi-arid conditions that apparently suited sauropods were more prevalent in the Southern Hemisphere during the Cretaceous [207]. In Laurasia, sauropods went extinct in North America in the earliest Late Cretaceous (§4*b*), and it is conceivable that this group struggled to compete for resources when faced with the advanced herbivorous adaptations acquired by ornithischians [65,207]. Several major ornithischian radiations (hadrosaurs, ankylosaurids, pachycephalosaurs and ceratopsids) occurred in Laurasia in the Late Cretaceous, after the disappearance of the key land connections with Gondwanan continents (figure 2; table 1): although some of these groups eventually reached Gondwana in the latest Cretaceous (e.g. hadrosaurs [155,160,195]), these biogeographic factors may have offered titanosaurs the ‘breathing space’ to diversify without facing severe competition [207].

Ephemeral land connections played an important role in facilitating intercontinental dinosaurian dispersals (§4*b*). Many of these were high-latitude routes, such as the Bering Strait landbridge between Laramidia and eastern Asia during the mid- and latest Cretaceous, the Maastrichtian De Geer landbridge linking Greenland and western Europe and the northern margin of Antarctica between South America and Australasia in the mid-Cretaceous [2,138,139,164] (figure 2; table 1). We would predict that theropods and ornithischians were able to cross high-latitude routes, but these would have been barred to sauropods (except perhaps under exceptional climatic conditions). There is good evidence that theropods and ornithischians (but not sauropods) dispersed via Beringia [2,104,138–142]. Similarly, southeast Australia’s cool temperate climate during the late Early Cretaceous probably discouraged sauropod occupation, and climatic barriers prevented dispersal from South America via Antarctica, explaining their absence in Australia 115−105 Ma [111,207,220,221]. However, global warming during the latest Albian–Turonian (*ca* 105−90 Ma) flattened the latitudinal thermal gradient and pushed warmer conditions southward, creating suitable habitats for sauropods along the South America–Australia dispersal route via the northern margin of Antarctica (figures 2 and 3*b*,*c*) and so giving rise to the rich sauropod fauna of the mid-Cretaceous of Queensland [111].

## 6. Challenges and opportunities

### (a) Challenges: problems with our data

Although some consensus has emerged, the study of dinosaurian biogeography retains many disagreements and uncertainties. These problems can be traced to several sources, but the most important ones are disagreements or a lack of resolution in key types of underpinning data (e.g. taxon identification, phylogenetic relationships, the ages of rocks or fossils and palaeogeographic or palaeoclimatic reconstructions), the patchiness of the fossil record and current limitations on analytical methods. Taxonomic and phylogenetic errors clearly have the potential to scramble biogeographic signals or even produce spurious ones. For example, although recent phylogenies have supported close biotic affinities between South American and Australian theropods, sauropods and ankylosaurs in the Late Cretaceous (§4*c*), ornithopod relationships are more equivocal [2,222–224]. As noted above, palaeoclimatology and palaeogeography are vital for understanding the causal factors underpinning biogeographic patterns, yet these fields are as prone to disagreement as any other area of deep-time research. For example, many aspects of the sequence and timing of the Late Jurassic–Palaeogene break-up of Gondwana are debated [2,111,116,145,162,181]. Moreover, finer-scale and ephemeral features such as landbridges generated by subtle changes in sea level can make the difference between inferences of geodispersal or trans-oceanic dispersal (e.g. [168,225]), and yet it is precisely these aspects of palaeogeography that are the most difficult to resolve accurately [2]. A wide array of geological and anthropogenic factors means that fossil record sampling is very far from complete, and extreme unevenness of coverage is the norm both temporally and spatially [62,226–232]. For example, as noted in §5, the palaeotropics are particularly poorly sampled for the Early and Middle Jurassic, but sampling improves greatly for the Cretaceous [207]. Similarly, the Cretaceous fossil record of South America has much better sampling than that of Africa, and the Early Cretaceous of Indo-Madagascar has yet to reveal any formations suitable for the preservation of dinosaurs ([2] and references therein). Missing data not only degrade biogeographic patterns but may also introduce distortions that give a false impression of past events [233,234]. For example, Upchurch [145] proposed that vicariance is a more fragile pattern than many dispersal or regional extinction scenarios and is thus more difficult to detect when sampling is inadequate. Moreover, some putative trans-oceanic dispersals might actually be instances of vicariance distorted by gaps in the stratigraphic ranges of taxa [2]. In short, the existence of large-scale spatio-temporal sampling biases suggests that some biogeographic inferences are likely to be more reliable than others, and an appreciation of their nature and magnitude is the first step to ameliorating their negative impacts (see below).

### (b) Opportunities: new data and methods

There has been a considerable influx of new data on dinosaurs during the past few decades (e.g. [235,236]), partly driven by fieldwork that has targeted biogeographically important or under-sampled regions such as Antarctica [204,215], Alaska [140,213], Australia [111,148,237,238] and Africa [115,154,155,167,178,181,195]. These new data, combined with ongoing database construction [231,239] and phylogenetic work, are filling some key gaps in our understanding of dinosaur biogeography, as illustrated by progress regarding Australian faunas (§4*c*). Clearly, new data are vital to resolving many macroevolutionary debates [232], and historical biogeography is no exception. However, appropriate analysis is also key: Upchurch [2] demonstrated that many of the older biogeographic approaches applied to dinosaurs were inadequate and generated errors and misconceptions. For example, a once popular method of mapping geographic areas onto cladograms as if they are character states (e.g. [21]) biases the results in favour of dispersal and eliminates any possibility of finding vicariance [2]. Fortunately, a suite of new analytical methods, based on dated phylogenies, have become available recently and have been applied to dinosaurs. These include maximum likelihood and Bayesian techniques for estimating ancestral area distributions [15,22,87,103,104,111,115,125,126,134,141,142,154,155,163,169,200,240–243]. There are also network biogeographic approaches that use phylogenetic distances to quantify the similarities or differences between biotas, and so construct metrics of endemicity or cosmopolitanism [76,133,179]. Such integrations of dated phylogenies into biogeographic analyses help at least partially fill some of the gaps in the fossil record and also provide greater rigour, quantification and repeatability. Furthermore, these various approaches are opening up rich opportunities for spatially explicit biogeographic modelling and exploration of the sensitivity of results to various assumptions and uncertainties (e.g. [11]).

Ecological niche modelling (ENM) and HSM are crucial tools in modern conservation, providing valuable insights into species distribution dynamics in response to abiotic changes. By integrating biotic (distribution) and environmental (climatic) data, ENM and HSM generate quantitative, multidimensional representations of the abiotic requirements of species. ENM and HSM are playing an increasingly important role in Mesozoic dinosaur palaeontology (e.g. [149,207]) and other deep-time studies demonstrating their potential in palaeobiogeography, macroecology and macroevolution [244–249]. For example, such quantification can reveal how climatic shifts influence palaeogeographic dispersal routes, barriers and filters (§§3 and 5). One long-standing problem with historical biogeographic analysis is that it has been impossible to distinguish between genuine absence (i.e. an organism did not occur in a given area) and pseudo-absence (i.e. the organism was present but has not been sampled) [2,106]. However, ENM-type methods could help us make more accurate diagnoses of genuine versus pseudo-absence. For example, a given rock formation might comprise sediments of a type which we know are capable of preserving dinosaurs, but the latter are absent [149]. Knowledge of the climate and habitat represented by this formation, combined with ENM, could reveal that dinosaurs were unlikely to have lived in that region, and thus, their absence is probably genuine. Thus, one exciting future prospect is that ENM-type methods, combined with databases that record both fossiliferous and non-fossiliferous sedimentary formations (e.g. [231]), have the potential to improve the quality of the data we feed into biogeographic analyses.

## 7. Conclusions and prospectus

The study of dinosaur biogeography requires a multidisciplinary research programme that incorporates fieldwork, databasing, taxonomy, phylogenetic analysis, tree dating, analytical biogeographic methods and palaeoclimatic and palaeogeographic information. A general outline of dinosaurian biogeographic history can now be established based on considerable data and rigorous analysis and includes a potential southern Gondwanan origin in the Carnian, northward dispersals during the Norian in response to climatic ameliorations, cosmopolitanism in the Early Jurassic as dinosaurs radiated to fill niches left vacant after the ETE and a complex reticulate Middle Jurassic–Cretaceous pattern created by waves of vicariance and geodispersal prompted by Pangaean fragmentation and the formation of ephemeral land connections. Many aspects of this scenario remain controversial, and one of the key areas to emerge concerns the impact of climate and how this may enhance or prohibit particular dispersal barriers or corridors. Problems also persist regarding the quantity and quality of data, and methods for measuring and ameliorating uneven sampling of the fossil record are in their infancy with regard to historical biogeography.

In terms of future prospects, clearly, we must continue to target the collection of data from critically under-sampled portions of the fossil record. It is also probable that there is a considerable amount of data in museums that has not yet made it into publications and databases [250]. Clarification of phylogenetic relationships is also key, as illustrated by the plethora of evolutionary trees, some of which appear to conform well to a given biogeographic scenario and others that do not. In particular, controversy surrounding the area of origin for dinosaurs is only likely to be resolved once the fundamental relationships between the major clades (theropods, sauropodomorphs and ornithischians) and other critical groups (e.g. herrerasaurians and silesaurids) have been firmly established. However, concerns regarding fossil record sampling and phylogenetic relationships apply generally to palaeobiological research, so it is also important to consider issues that are specific to progress in historical biogeography. One key line of research is likely to be the appropriate incorporation of missing data or other measures of spatio-temporal sampling into the phylogenetic biogeographic methods outlined in §6*b*. We will also need to explore whether these disparate methods, when applied to the same data, yield the same conclusions. If not, then why not? A second key theme is modelling the dispersal abilities of various dinosaurian groups. The above discussion of whether the earliest dinosaurs were restricted to cooler, humid mid-palaeolatitudes by arid palaeotropics or were, in fact, already equipped with the adaptations required for dispersal illustrates how behavioural, ecological, biomechanical and physiological features may be key to understanding biogeographic distributions. A similar argument can be made regarding transoceanic dispersal: it seems likely that some dinosaurs crossed marine barriers, but what were the limits on distance and endurance placed on such dispersals by biomechanical and physiological constraints? In the future, it will be both interesting and important to model the trans-oceanic dispersal abilities of various dinosaurs and build this information into the dispersal constraints used in ancestral area estimations and spatially explicit biogeographic studies. Recently, researchers have started to look at these issues in the context of spatially explicit models of trans-oceanic dispersal in mammals [225,251], and it may be that similar approaches can be adapted for use with dinosaurs. Thus, while many fundamental questions remain unanswered or controversial, it seems that we are now approaching the large datasets and analytical methods required to untangle the complex biogeographic history of dinosaurs.

## Data Availability

This article has no additional data.
